# Eliminating material constraints for nonlinearity with plasmonic metamaterials

**DOI:** 10.1038/ncomms8757

**Published:** 2015-07-21

**Authors:** Andres D. Neira, Nicolas Olivier, Mazhar E. Nasir, Wayne Dickson, Gregory A. Wurtz, Anatoly V. Zayats

**Affiliations:** 1 Department of Physics, King's College London, Strand, London WC2R 2LS, UK

## Abstract

Nonlinear optical materials comprise the foundation of modern photonics, offering functionalities ranging from ultrafast lasers to optical switching, harmonic and soliton generation. Optical nonlinearities are typically strong near the electronic resonances of a material and thus provide limited tuneability for practical use. Here we show that in plasmonic nanorod metamaterials, the Kerr-type nonlinearity is not limited by the nonlinear properties of the constituents. Compared with gold's nonlinearity, the measured nonlinear absorption and refraction demonstrate more than two orders of magnitude enhancement over a broad spectral range that can be engineered via geometrical parameters. Depending on the metamaterial's effective plasma frequency, either a focusing or defocusing nonlinearity is observed. The ability to obtain strong and fast optical nonlinearities in a given spectral range makes these metamaterials a flexible platform for the development of low-intensity nonlinear applications.

## Abstract (web summary)

Nonlinear optical properties of conventional materials have limited spectral tuneability as they are defined by the material properties themselves. Here, the authors demonstrate that strong nonlinearity can be achieved in metamaterials where negligible nonlinearity of the constituent materials exists.

## Graphical abstract

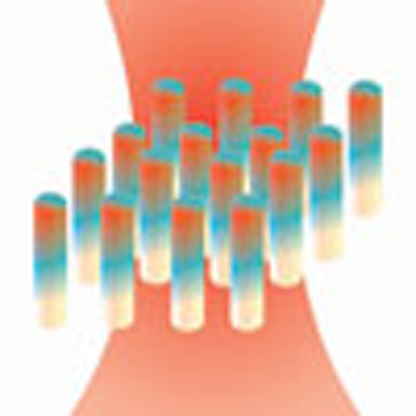

Plasmonic materials, capable of supporting surface plasmon excitations due to the coupling of light to free electrons, provide some of the highest and fastest nonlinearities compared with semiconducting or transparent dielectric materials^1^,^2^,^3^,^4^,^5^. The strong absorption of optical energy, characteristic of metals, allows an efficient excitation of free electrons leading to a high third-order (Kerr type) optical nonlinearity, which arises from a modification of the electron temperature due to non-equilibrium electron population created by the absorbed light in the conduction band^3^,^4^,^5^,^6^,^7^,^8^. This Kerr-type nonlinearity is expressed as changes in the real and imaginary parts of the refractive index and manifests itself as nonlinear refraction and absorption, respectively. The latter corresponds to the intensity-dependent variation of the material's absorption and the former to the intensity-dependent variation of the phase of the wave as it propagates through the material, leading to focusing (positive nonlinearity) or divergence (negative nonlinearity) of the optical field^1^.

For plasmonic materials, both nonlinear absorption and refraction are usually stronger than those of the typical reference nonlinear medium, CS_2_, and typical semiconductors such as Si or GaAs^4^,^9^,^10^,^11^. The strong Kerr nonlinearity of metals is significantly restricted to the spectral range of the interband electronic transitions where efficient electron excitation in the conduction band takes place, leading to the strongest nonlinear response. This nonlinearity becomes weaker at frequencies away from the interband absorption, limiting its usefulness to the (near) ultraviolet spectral range of control light wavelength. On the other hand, the strong absorption near the interband resonance, in many cases, prohibits useful applications when the signal light, which may be controlled by this nonlinearity, overlaps this spectral range.

Plasmonic metamaterials allow engineered light–matter interactions mediated by the plasmonic resonances of their subwavelength constituent elements (‘meta-atoms')^12^,^13^,^14^. Different metamaterial realizations have been proposed to design the refractive index and optical properties throughout visible and telecom wavelength range including negative refraction^15^ and hyperbolic dispersion^16^,^17^. These properties have led to the development of numerous applications in sensing^18^,^19^,^20^,^21^, Purcell factor engineering^22^ and all-optical modulation^23^,^24^,^25^,^26^. A large nonlinear optical response has been predicted and observed for controlling metamaterials' transmission, reflection and polarization properties with light^27^,^28^,^29^,^30^. In most of these approaches, the nonlinear response of the plasmonic metal induced by the interband absorption of a control light was used, and a change of refractive index, strongly diminished away from these transitions, was detected using the metamaterial's ability to act as a sensitive refractive index sensor.

In this work, we go beyond the nonlinearity related to interband transitions in plasmonic metals and show that the plasmonic nanorod metamaterial geometry provides strong nonlinearity, larger than the interband nonlinearity of the plasmonic metal itself, at any *a priori* designed wavelength. We directly measure the third-order nonlinearity of the metamaterial using a femtosecond z-scan technique and show that a positive (focusing) or negative (defocusing) nonlinearity can be achieved by engineering the metamaterial nanostructure in the desired spectral range. The observed enhancement is almost two orders of magnitude larger compared with unstructured Au under interband excitation over a broad spectral range. This opens up opportunities for the on-demand engineering of strong, ultrafast free-electron-based nonlinearities in macroscopic metamaterials.

## Results

### Linear optical properties

**Figure 1:**
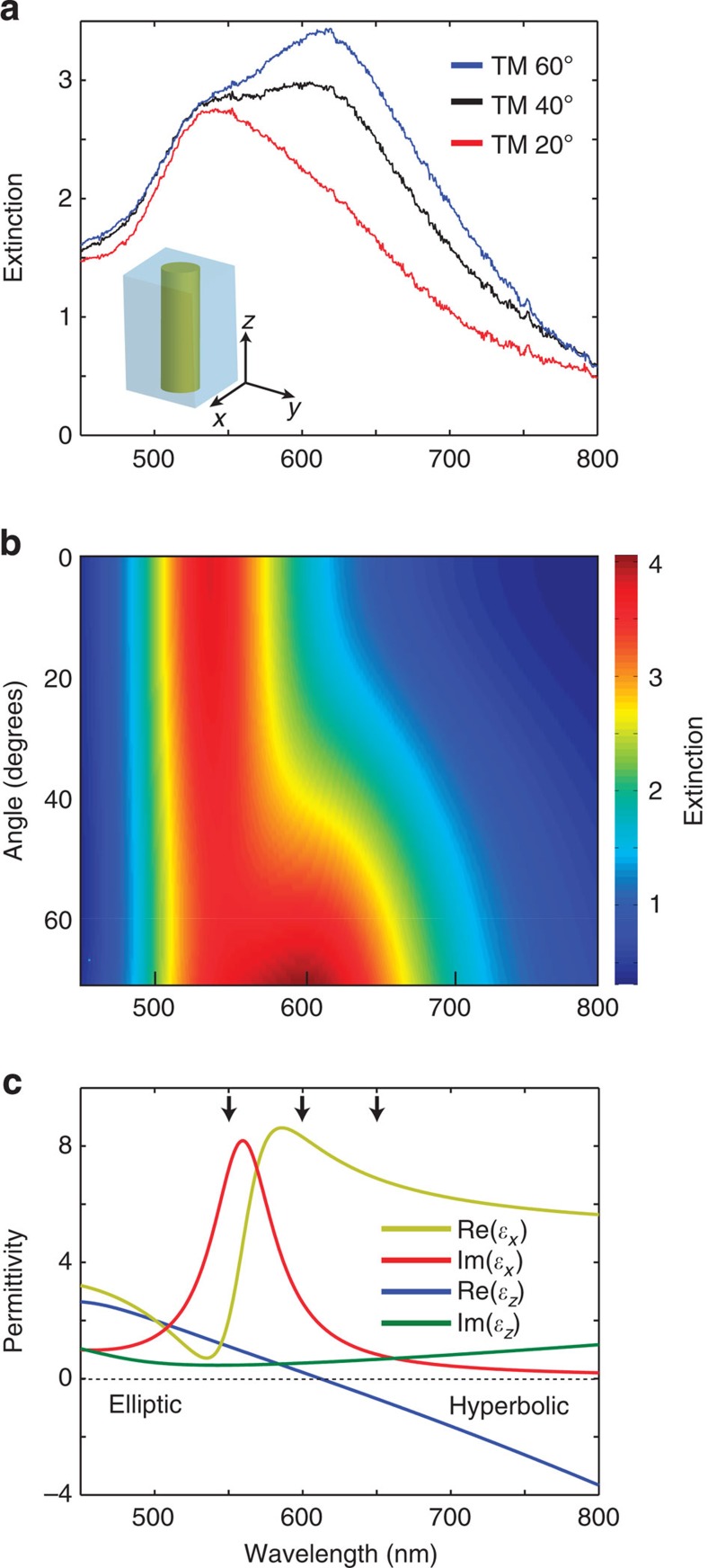
Plasmonic nanorod metamaterial properties. (**a**) Extinction spectra of the nanorod metamaterial measured at different angles of incidence. (**b**) Simulated extinction dispersion of the metamaterial. (**c**) Effective permittivities of the metamaterial deduced from the effective medium model. Arrows indicate the wavelengths where the nonlinear coefficients were measured.

The Au nanorod metamaterial (see Methods for the details of fabrication) displays the typical polarization-dependent transmission resonances (Fig. 1). This behaviour can be reproduced by simulations using an anisotropic permittivity tensor (see Methods for the details on theoretical description and modelling) having diagonal components describing the response to the field polarized perpendicular (*ɛ*_*x*_=*ɛ*_*y*_) and along (*ɛ*_*z*_) the nanorod axis (Fig. 1b). The extinction spectra exhibits two main features: a resonance at a wavelength of ∼550 nm, which has a spectral position invariant with the angle and polarization of the incident light, originates from the interaction of the plasmonic resonances of the nanorods excited perpendicular to their axes, and a resonance at around 600 nm, which can only be excited by light having an electric field component parallel to the nanorod axis and is therefore dependent on the angle of incidence and polarization. The latter arises near the effective plasma frequency of the metamaterial, at which the crossover from elliptic to hyperbolic dispersion regime takes place as Re(*ɛ*_*z*_) transitions from positive to negative values. This frequency can be designed by changing either the geometrical parameters or the plasmonic and dielectric constituents of the metamaterial (see Methods).

### Nonlinear optical properties

**Figure 2:**
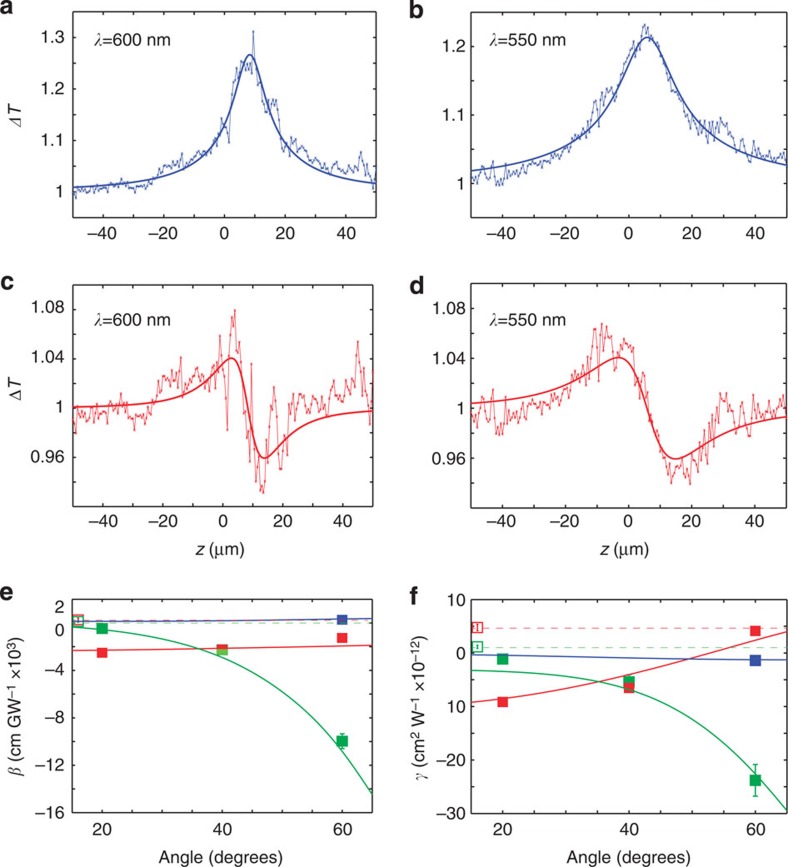
Nonlinear measurements. (**a**,**b**) Open and (**c**,**d**) closed aperture z-scan measurements (thin lines) and fit (thick lines) at wavelengths near (**a**,**c**) the effective plasma frequency and (**b**,**d**) the Au interband transitions. (**e**) Nonlinear absorption and (**f**) nonlinear refractive index dependence on the angle of incidence for different wavelengths: (squares) experiment, (lines) simulations. Red, 550 nm; green, 600 nm; and blue, 650 nm. Empty squares and dashed lines are for a smooth Au film. All measurements and simulations are for TM-polarized incident light. The errors bars in **e** and **f** are smaller than the size of the square unless indicated.

The third-order nonlinearity of the metamaterial was studied at different angles of incidence and different wavelengths using a femtosecond z-scan technique. Both open and closed aperture measurements (Fig. 2a–d) were performed in a broad spectral range (550–650 nm), covering both the elliptic and hyperbolic regimes of the metamaterial's dispersion, and at different angles of incidence. Similar z-scan measurements were performed in the same conditions for a smooth 50-nm-thick Au film for comparison.

The z-scan allows direct determination of the nonlinear refraction, *γ*, and absorption, *β*, coefficients related to the intensity-dependent, complex effective refractive index of the metamaterial 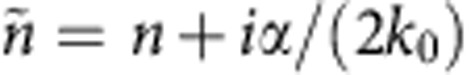, *k*_0_ being the light wave vector and 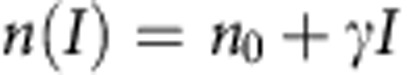 and 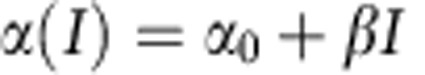, where *n*_0_ and *α*_0_ are the linear refractive index and absorption, respectively, and *I* is the intensity of the incident light (see Methods for details). As a consequence of the metamaterial's anisotropy, the effective refractive index is angular dependent since 
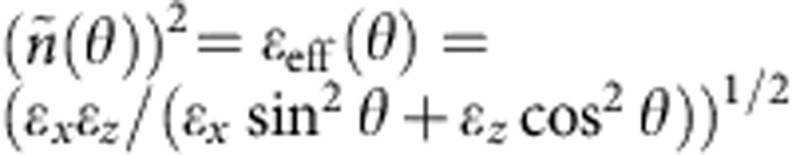
, where *θ* is the angle of propagation within the metamaterial with respect to the nanorod axis. Thus, the retrieved *γ*(*θ*) and *β*(*θ*) are the effective nonlinear refraction and absorption coefficients corresponding to the variation of the metamaterial's effective linear refractive index for a specific angle of incidence that can be related to the components of the effective third-order nonlinearity tensor of the anisotropic metamaterial (see Methods).

The nonlinear coefficients are very different for a smooth Au film and the Au-based metamaterial both in the value and sign of the nonlinearity (Fig. 2e,f). As expected from their definition, the nonlinear coefficients of the metamaterial are strongly angular dependent, which is especially pronounced near a wavelength of 600 nm, corresponding to the effective plasma frequency. For wavelengths in the elliptic dispersion range (*λ*<600 nm), nonlinear absorption is almost constant, whereas nonlinear refraction that is defocusing for smaller angles, decreases with the incidence angle and changes sign becoming focusing one. In the so-called epsilon near-zero (ENZ) regime with Re(*ɛ*_*z*_)∼0 at around 600 nm, both *β* and *γ* markedly increase with the incident angle. The largest nonlinearity was experimentally measured in these conditions at an angle of incidence of 60° and at a wavelength close to the effective plasma frequency of the metamaterial: *γ*≈−2.4 × 10^−11^ cm^2^ W^−1^ and *β*≈−9967, cm GW^−1^. In comparison, the measured nonlinear coefficients for a smooth 50-nm-thick Au film sharply decrease with increasing wavelength away from the interband transitions with the experimentally measured values *γ*≈4.8 × 10^−12^ cm^2^ W^−1^ and *β*≈272 cm GW^−1^ at 550 nm wavelength and *γ*≈1.2 × 10^−12^ cm^2^ W^−1^ and *β*≈122 cm GW^−1^ at 600 nm wavelength. These values for a smooth Au film are in agreement with those obtained in a previous study using femtosecond pulse excitation^4^. At the same wavelengths, *γ* and *β* of the nanorod metamaterial are approximately 20 and 100 times larger than those measured for a smooth Au film. Surprisingly, the maximum value obtained for *γ* and *β* for the metamaterial away from Au interband transitions is approximately 5 and 40 times larger than the maximum values measured for a smooth gold film close to the interband transition where they are highest. While in the studied range of frequencies, the nonlinearity of smooth Au is always positive (induced absorption and focusing), the Au nanorod metamaterial can provide either induced absorption, transparency, focusing or defocusing nonlinearity, depending on the combination of light wavelength and angle of incidence. Thus, not only the strength but also the sign of the nonlinearity can be designed.

## Discussion

On the basis of the above experimental observations, the dominating contributions of the effective third-order susceptibility components can be identified (see Methods):


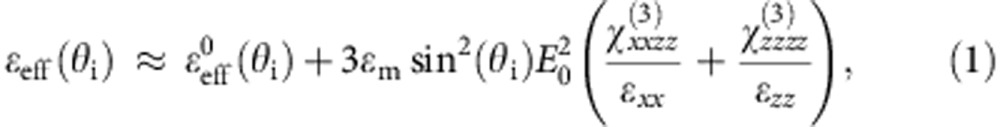


where 
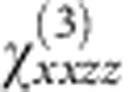
 and 
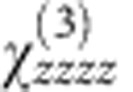
 are the components of the third-order nonlinear susceptibility tensor of the metamaterial, dominant for the incident transverse magnetic (TM) light, *ɛ*_m_ is the permittivity of the medium adjacent to the metamaterial where the incident wave is coming from, *θ*_i_ is the angle of incidence, and *E*_0_ is the incident electric field amplitude. As seen from equation (1), the linear anisotropy of the metamaterial can strongly enhance the nonlinear response, particularly when the linear permittivity tensor components become small, as in the case of the ENZ regime.

**Figure 3:**
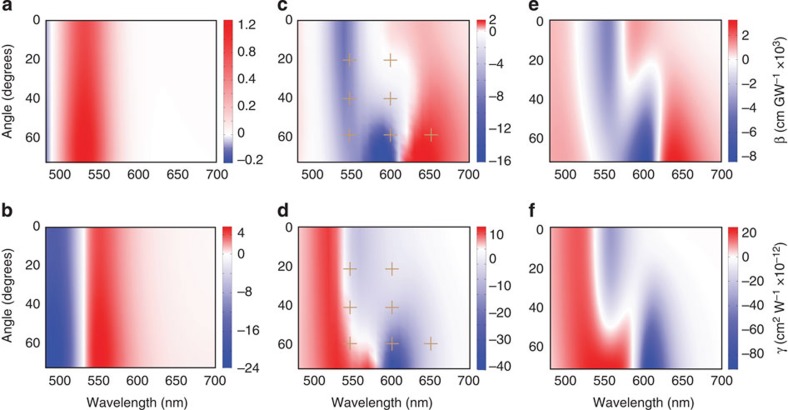
Nonlinear response model. Nonlinear absorption and refraction coefficients for a smooth gold film and the nanorod array metamaterial calculated using finite element method (**a**–**d**) and the effective medium theory (**e**,**f**): (**a**,**c**,**e**) nonlinear absorption and (**b**,**d**,**f**) nonlinear refractive index simulated for (**a**,**b**) the smooth 50-nm-thick gold film and (**c**–**f**) the nanorod metamaterial. The crosses indicate the points where the experimental data are presented in Fig. 2.

To understand the nonlinear response of the metamaterial, full-vectorial numerical simulations of the nonlinear optical processes were performed taking into account the internal structure of the metamaterial and the change in the Au permittivity on absorption of the energy from the optical excitation pulse (as described in Methods). We consider that the excitation pulse is absorbed by the metallic nanorods comprising the metamaterial due to the interband or intraband transitions, depending on the excitation wavelength (the anodic alumina oxide (AAO) matrix is considered as non-absorbing). This induces an imbalance between the electron and lattice temperature in the metal nanorods. The excited electrons then relax back to the ground state as a consequence of energy exchange via electron–electron and electron–phonon scattering. Taking into account these processes, the electron temperature variations under the excitation pulse can be calculated depending on its energy and duration, and, thus, the intensity-dependent effective refractive index of the metamaterial can be evaluated, in turn allowing the effective nonlinear coefficients *γ* and *β* to be deduced under different excitation wavelengths and incident angles. To validate the model, the nonlinearity of a smooth 50-nm-thick Au film was also simulated and provided excellent agreement with the experimental data (Fig. 3a,b), showing a high nonlinearity at wavelengths shorter than 560 nm near the interband transition of gold. These values are also in a good agreement with the known values of third-order nonlinearity of Au films for similar pulse durations^4^.

The simulations for both the smooth film and the metamaterial provide excellent agreement with the measured data in terms of both the observed trends and absolute values of the nonlinear coefficients. Near the metamaterial's absorption resonance (∼550 nm), *β* is constant and negative for all angles of incidence (Fig. 3c), whereas *γ* decreases, changing its sign from negative to positive at a given angle (Fig. 3d). On the other hand, near the effective plasma frequency, both *β* and *γ* are negative and increase with increasing angle of incidence. It should be noted that the simulations using the nonlinear transfer matrix method and effective medium description of the metamaterial, which does not take into account nonlocal effects^31^ or the inhomogeneous distribution of the electron temperature in the nanorods, describe similar spectral and angular behaviour but with underestimated values of the nonlinear coefficients (Fig. 3).

**Figure 4:**
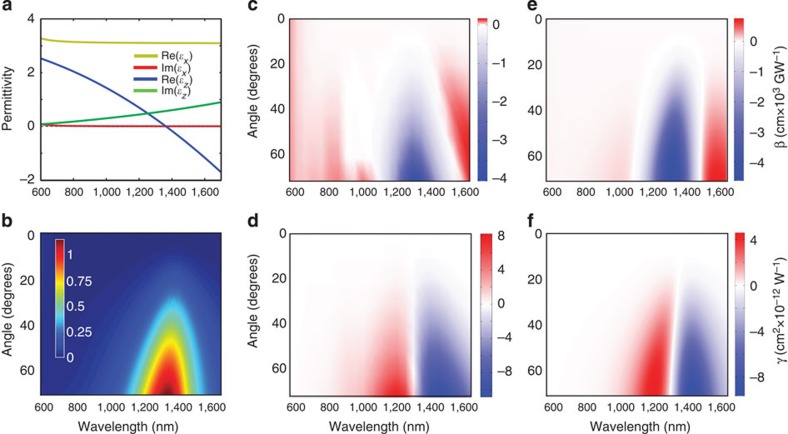
Tuning the third-order nonlinearity of the metamaterial. (**a**) Effective permittivity spectra of a nanorod metamaterial designed with the effective plasma frequency at around *λ*=1.35 μm (ENZ regime at the telecommunication spectral range). (**b**) Linear extinction dispersion of the metamaterial in **a**. Nonlinear absorption (**c**,**e**) and refraction (**d**,**f**) coefficients calculated using finite element method (**c**,**d**) and EMT (**e**,**f**) of the metamaterial with the parameters as in **a**. Metamaterial consists of Au nanorods of 150-nm height and 17-nm diameter arranged in a square array of 95-nm period embedded in AAO matrix.

The highest third-order nonlinear response has been observed near the ENZ conditions, corresponding to the effective plasma frequency of the metamaterial^32^. This frequency can be tuned as desired by engineering the metamaterial's geometrical structure with the same constituent materials, and therefore a strong nonlinear response can be achieved at the wavelengths where the constituent materials have negligible nonlinearity. As an example, a nonlinear metamaterial has been designed for the telecommunication spectral range 1–1.6 μm in which Au has negligible nonlinear response (Fig. 4). This has been accomplished by adjusting only the nanorod diameter to achieve an effective plasma frequency at a wavelength of approximately 1.35 μm. Interestingly, despite significantly lower absorption, the observed nonlinear refraction and absorption are only three and five times smaller, respectively, than those of the metamaterial designed for the visible spectral range where the nonlinearity of Au is much stronger. The spectral and angular dependencies of the nonlinear response are similar to those for the visible spectral range, determined by the effective plasma frequency (cf. Figs 3 and 4).

In the ENZ and hyperbolic regimes, the dependence on the angle of incidence is significant since the *ɛ*_*z*_ component plays a dominating role (Figs 3 and 4). In contrast, in the elliptic regime, the nonlinearity is mainly governed by the value of Re(*ɛ*_*x*_) and, therefore, the respective nonlinear absorption and refraction are almost invariant with the incident angle. A strong nonlinearity, either focusing (*γ*>0) or defocusing (*γ*<0) can be designed using the metamaterial irrespective of the type of nonlinearity of the constituent materials and the operating wavelength (Figs 3 and 4). As can be observed, the sign of the nonlinear refraction coefficient changes while crossing the effective plasma frequency, providing positive nonlinearity at shorter wavelength (elliptic regime) and negative nonlinearity at longer wavelength (hyperbolic regime). At the same time, the nonlinear refraction (*γ*) of Au is positive below the interband transition (*λ*>550 nm), while in the case of the metamaterial this coefficient is now negative and becomes positive only at large angles of incidence for wavelengths shorter than that of the effective plasma frequency (Figs 3c,d and 4c,d) reaching an experimentally measured maximum negative value of *γ*≈−2.38 × 10^−11^ cm^2^ W^−1^ and a maximum positive value of *γ*≈4.1 × 10^−12^ cm^2^ W^−1^. In the case of nonlinear absorption, which is always positive in the spectral range below the interband transitions for a smooth Au film, a strong induced nonlinear transparency (*β*<0) is observed in the elliptic dispersion range, yet strong induced absorption (*β*>0) is present in the hyperbolic regime.

Looking beyond nonlinearities of plasmonic metals, nonlinear transparent dielectrics, such as beta barium borate, lithium triborate or lithium niobate exhibit a weak and broadband third-order nonlinear susceptibility throughout visible and telecom spectral range, which is typically smaller than the nonlinearity of Si at telecom wavelengths^33^. These nonlinearities are typically more than three orders of magnitude smaller than the one observed for Au nanorod-based metamaterials. Semiconductor materials, such as GaAs, exhibit higher nonlinearity which near interband transitions, is only one order of magnitude lower than these metamaterials in a similar spectral range^11^. An interesting example is graphene, which provides a nonlinear response similar to the plasmonic metamaterial in the infrared and the visible^34^. Indeed, graphene and transparent dielectrics provide a broadband nonlinear response throughout the visible and infrared spectral range, with semiconducting materials exhibiting a more narrowband nonlinearity limited to the interband transition wavelength range. The nonlinear response of the nanorod metamaterial (Figs 3 and 4) has also a broader spectral range than typical for semiconductors and metamaterials based on split-ring resonators.

The enhanced nonlinearity of nanorod metamaterials arises from the specific dispersion of plasmonic metamaterials that results in vanishingly small permittivity components (equation (1)). Such hyperbolic metamaterials can in principle also be designed using highly doped semiconductors in certain frequency ranges and, thus, the nonlinearity of semiconductors can be harnessed via a similar mechanism as in the metamaterials described here. ENZ-enhanced nonlinearities have been recently measured via third-harmonic generation spectroscopy (which is related to third-order susceptibility components) in natural (unstructured) ENZ material indium tin oxide^35^. Similarly, an enhancement of the third-harmonic generation efficiency has been observed in Si nanodisks due to magnetic dipole resonance excitation in individual disks^36^. While the underpinning mechanisms are different than in the discussed nanorod metamaterials, these nonlinearities can be used for designing nonlinear metamaterials to further enhance their nonlinear response and achieve further spectral tuneability.

In conclusion, we have shown that the enhanced focusing (*γ*>0) or defocusing (*γ*<0) nonlinearity and associated induced absorption (*β*>0) or transparency (*β*<0) of Au nanorod-based metamaterials can be designed irrespective of the type of nonlinearity of the constituent materials and their resonant nonlinear response. Thus, strong nonlinearity can be achieved in metamaterials at wavelengths where negligible nonlinearity of the constituent materials exists. Since the temporal response of plasmonic nonlinearities are on the order of few hundreds femtoseconds depending on wavelength and absorbed power, they allow the possibility to modulate light at ultrafast rates exceeding 1 THz^2^,^5^,^23^,^25^. Furthermore, this nonlinearity can be tuned in a broad range of wavelengths through the design of the metamaterial's dimensions. Therefore, the metamaterial can be suitable for spectrally demanding applications, including those at telecom wavelengths, removing the conventional reliance on the electronic transition resonances of conventional nonlinear materials, and providing the freedom to design strongly nonlinear materials at any frequency of interest and choose the sign of the nonlinearity to achieve either focusing or defocusing of light. While optical absorption of plasmonic metamaterials is higher than for dielectric or semiconductor nonlinear materials due to presence of metal, the observed ultrafast nonlinearities that are stronger by three to four orders of magnitude are advantageous for nano- and microscale nonlinear components, which can be integrated into Si photonic circuitry or optical fibres providing designer nonlinear functionalities.

## Methods

### Sample preparation

Plasmonic nanorod metamaterials were fabricated via Au electrodeposition into nanoporous AAO templates on a glass substrate. An Al film of 800 nm thickness was deposited on a substrate by magnetron sputtering. The substrate comprises a glass cover slip with a 10-nm-thick adhesive layer of tantalum pentoxide and a 7-nm-thick Au film acting as a weakly conducting layer. Highly ordered, nanoporous AAO was synthesized by a two-step anodization in 0.3 M oxalic acid at 40 V. After an initial anodization process, the porous layer formed was removed by etching in a solution of H_3_PO_4_ (3.5%) and CrO_3_ (20 gl^−1^) at 70 °C. An ordered, patterned surface was obtained after removal of the porous layer formed during first step of anodization. Then, the samples were anodized again under the same conditions as in the first step. The anodized AAO was subsequently etched in 30 mM NaOH to achieve pore widening and remove the barrier layer. Gold electrodeposition was performed with a three-electrode system using a non-cyanide solution. The length of nanorods was controlled by the electrodeposition time. The topography variations of the top surface of the samples are below 10 nm, similar to other samples fabricated with the same approach^32^. The nanorod array parameters used in this work are about 150-nm height, 50-nm diameter and 95-nm period. For comparison, Au films of 50 nm thickness deposited using magnetron sputtering on similar substrates were measured.

### Z-scan measurements

An amplified Ti:sapphire femtosecond laser was used to pump an optical parametric amplifier to achieve light pulses in a wavelength range between 500 and 750 nm. These pulses are sent first to a prism-based pulse compression system, which induces negative dispersion to compensate for the dispersion in both the optical parametric amplifier and the experimental setup. A 0.28 numerical aperture objective lens focuses the 50-fs pulse to a spot having a radius of ∼1.5 μm on the sample. The peak power at the focus was ∼80 GW cm^−2^. The metamaterial is then scanned across the focus of the beam over a 100-μm range (Supplementary Fig. 1). The fluencies used are within the range used in the other studies of similar nanorod metamaterials^17^ and the measured signals were repeatable and reversible in all instances, thus the damage threshold has not been reached in the experiments. The transmission through the sample is then determined from the measurement of the intensities incident on two InGaAs photodiodes having a bandwidth range from 500 to 1,700 nm, for every position of the sample. The z-scan measurements were performed in the so-called closed and open aperture regimes by opening or closing the aperture after the sample^9^. The measurements were performed using either s- or p-polarized light chosen by rotating a half-wave plate. The measurements were taken in pairs at low and high light intensities, and the normalization between these two measurements was performed to average out possible variations of linear transmission (for example, due to roughness or defects) when a sample is z-scanned^9^.

### Nonlinear coefficients retrieval from z-scan measurements

The intensity-dependent refractive index *n*(*I*) and absorption *α*(*I*) are defined as


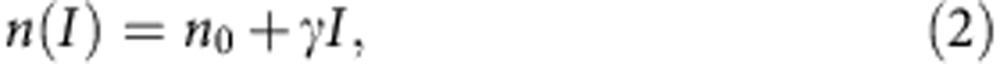



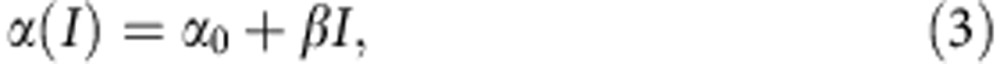


where *n*_0_ and *α*_0_ are the linear refractive index and absorption, respectively, *β* and *γ* are the nonlinear refraction and absorption coefficients, and *I* is the intensity of incident light. The coefficients *γ* and *β* are related to the real and imaginary part of the third-order nonlinear susceptibility *χ*^(3)^ through


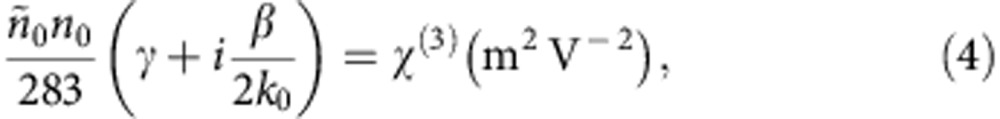


where 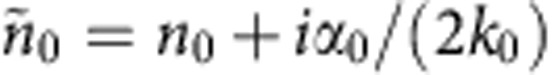 is the complex refractive index and 
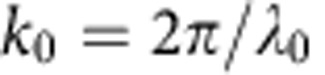
 is the wave vector related to the wavelength in vacuum (*λ*_0_). As a consequence of the metamaterial's anisotropy, the retrieved *γ* and *β* are the effective nonlinear refraction and absorption corresponding to the variation of the metamaterial's effective linear refractive index for a specific angle of incidence.

In the open aperture regime (Fig. 2a,b), all the transmitted intensity through the metamaterial is measured. Thus, variations in the transmission only correspond to the nonlinear absorption of the metamaterial, being strongest at the focus and smallest away from it. A positive peak-shaped dependence corresponds to a negative value of *β* (nonlinear transparency).

In the close aperture regime (Fig. 2c,d), only part of the intensity transmitted through the metamaterial is measured in a specific direction. Thus, variations in the transmission correspond to both nonlinear absorption and self-focusing or defocusing caused by the nonlinear refraction. Therefore, in the closed aperture regime, the transmission variations depend on both *β* and *γ*. A dependence solely determined by *γ* can be obtained by normalizing the close aperture curve with the open aperture curve obtained before. The curves in Fig. 2c,d correspond to a self-divergence of the transmitted beam related to a negative value of *γ* (defocusing nonlinearity).

Assuming a beam with a Gaussian spatial profile transverse to the scanning direction, the transmission in the case of open aperture is:


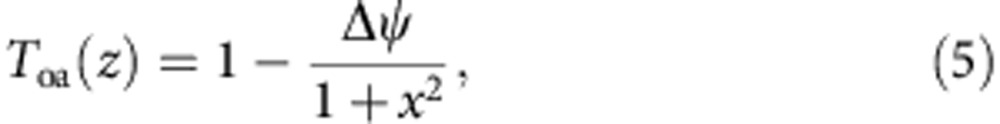


where 
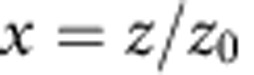
 and 
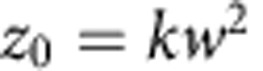
 with 
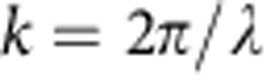
, and 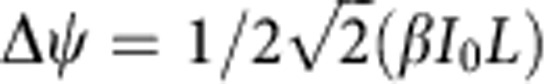 with *I*_0_ being the peak intensity at the metamaterial's surface and *L* is the sample thickness. In the case of close aperture, the transmission after the aperture is


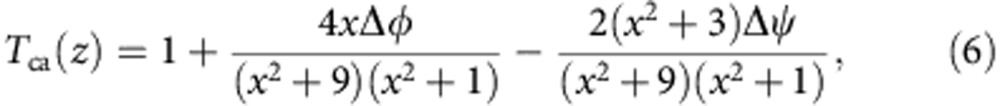


where 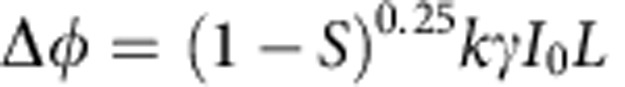 with *S* representing the transmission through the aperture. For very small apertures 
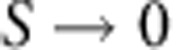
, this expression can be simplified as 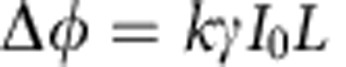. Therefore, by fitting the experimentally obtained open and close aperture z-scan transmission profiles with equations (5) and (6), the nonlinear parameters *γ* and *β* can be found^1^.

The complex effective third-order nonlinear susceptibility obtained from the measured values of *β* and *γ* is *χ*^(3)^≈(0.18–1.48*i*) × 10^−17^ m^2^ V^−2^ at a wavelength of 550 nm and an angle of incidence of 20°, and *χ*^(3)^≈(0.28–1.48*i*) × 10^−16^ m^2^ V^−2^ at a wavelength of 600 nm and an angle of incidence of 60°, compared with a smooth 50-nm-thick Au film, which is *χ*^(3)^≈(−0.09+1.19*i*) × 10^−19^ m^2^ V^−2^ and *χ*^(3)^≈(−0.5+1.08*i*) × 10^−19^ m^2^ V^−2^ at wavelength of 550 and 600 nm, respectively, in agreement with known values for smooth gold measured in this range of pulse duration^4^. The metamaterial's nonlinear susceptibility is up to three orders of magnitude larger than that for gold films, with a maximum theoretical value *χ*^(3)^≈(0.57–3.2*i*) × 10^−16^ m^2^ V^−2^ at a wavelength of 600 nm obtained for the metamaterial at an angle of incidence of 70°.

### Modelling the optical response of the metamaterial

Anisotropic metamaterials based on nanorod arrays can be described through the effective medium theory with a diagonal anisotropic permittivity tensor


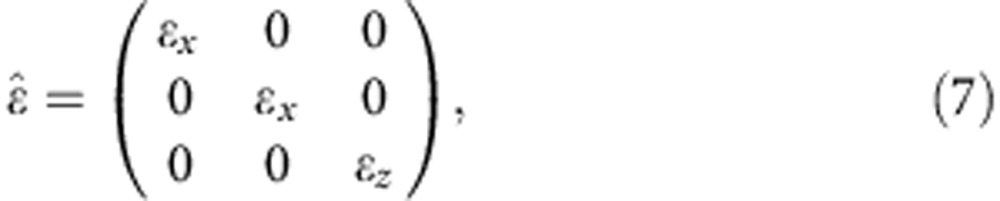


where the subscripts *x* and *z* indicate the components perpendicular and parallel to the nanorod axis, respectively. The components of this tensor are the function of the nanorod dimensions and can be evaluated through the effective medium theory^37^. These are plotted in Fig. 1c for the metamaterial studied.

For a plane wave propagating within the metamaterial at an angle *θ* to the nanorod axes (**z** direction) and polarized perpendicular to them (TE polarization), the effective permittivity of the nanorod array is represented by *ɛ*_*x*_. However, if the light has an electric field component along the nanorod axis (TM polarization), the electric field of the wave propagating within the metamaterial is **E**=*E*_*x*_**i**+*E*_*z*_**k**=*E*_0_ cos *θ***i**+*E*_0_ sin *θ***k** , where *E*_0_ is the amplitude of the electric field. Therefore, the effective permittivity depends on *θ* and is determined through^38^


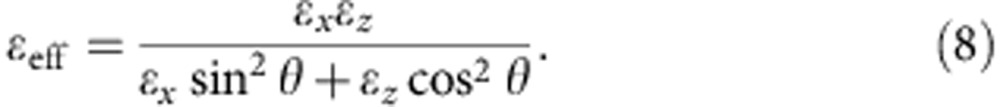


The effective permittivity components can derived from the Maxwell Garnet effective medium approximation for a given set of metamaterial's geometrical parameters as^37^


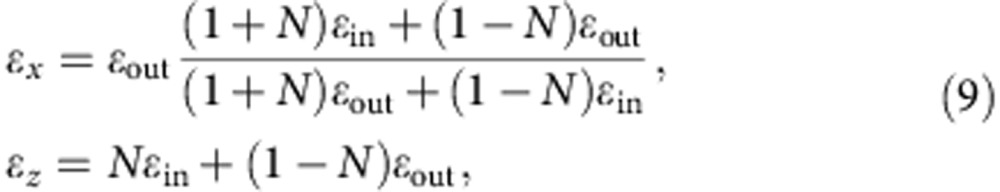


where *ɛ*_out_ and *ɛ*_in_ are the permittivities of the matrix (in this case an AAO matrix having permittivity of 2.89) and of the nanorods (the intensity-dependent permittivity of Au), respectively, and *N=πr*^2^/*p*^2^ is the concentration of nanorods defined via their radius *r* and period *p* of the array. The transfer matrix method^39^ was used to model the transmission spectra and plot the extinction dispersion (Fig. 1b). The modelled dispersion is in a good agreement with the experimental measurements as well as the dispersion numerically simulated using finite element modelling taking into account microscopic structure of the metamaterial.

### Modelling the intensity-dependent permittivity of Au

The gold permittivity is modelled within the random phase approximation that includes the dependence of the electron scattering on both electron (*T*_e_) and lattice (*T*_L_) temperatures^8^. Within this approximation, the intensity-dependent permittivity of Au can be described as the sum of the intraband permittivity and interband permittivity *ɛ*=*ɛ*_inter_+*ɛ*_intra_, which are dependent on the electron's intraband (transitions within the conduction band) and interband (transitions from the *d*-band to the conduction *sp*-band) transitions, respectively. The latter term can be reduced to a Drude-like model as


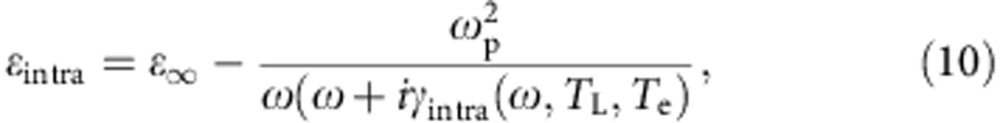


where *ω*_p_=2.168 × 10^15^ rad s^−1^, *ɛ*_∞_∼1 is the high-frequency limit of the permittivity, and *γ*_intra_(*ω*,*T*_L_,*T*_e_) is due to both electron–electron and electron–phonon scattering. The interband contribution can be expressed as^6^,^40^


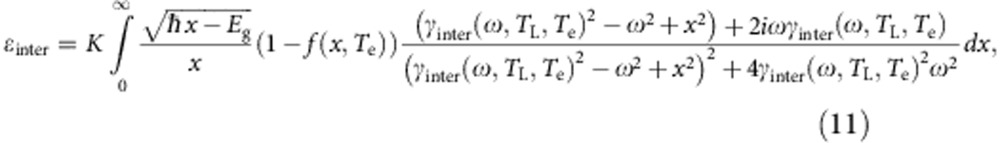


where 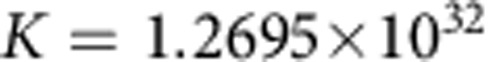 is the wave vector independent constant reflecting the strength of the transition dipole moment for interband transitions, *E*_g_=1.98 eV is the transition energy between the *d*- and the *sp*-bands, *f*(*x*,*T*_e_) is the Fermi–Dirac distribution for the electrons at an equilibrium temperature *T*_e_ and depends on the Fermi level *E*_f_=2.43 eV and the quasiparticle (electron-hole) scattering for interband transitions *γ*_inter_(*ω*,*T*_L_,*T*_e_), which also depends on electron and lattice temperatures. In addition, as a consequence of the nanorods fabrication procedure, the mean free path of electrons is reduced from the bulk value *L* to a restricted value *R* using the following correction term^38^


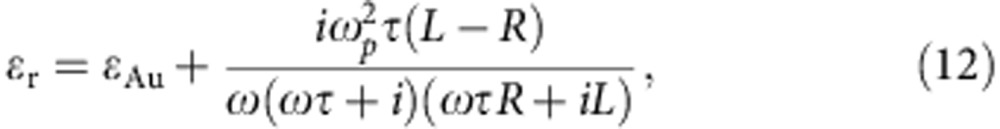


where *ɛ*_Au_ is the permittivity of bulk gold obtained from the sum of equations (10) and (11), *L*=35.7 nm and *R*=15 nm is derived to fit the measured extinction shown in Fig. 1b. For the simulations of the nonlinear response of a sputtered thin Au film, *R*=*L* was used to recover the bulk Au permittivity.

The steady-state electron temperature reached in the nanorods as a result of optical absorption of the transmitted beam is calculated from the energy stored by the electrons *E*_s_(*t*), under the optical pulse excitation with an input intensity *I*_i_(*t*). Therefore, the variation in the stored energy is proportional to the energy absorbed by the nanorods in a given interval d*t*:


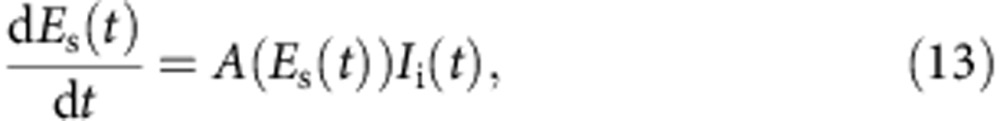


where *A*(*E*_s_(*t*)) is the sample absorption and depends on the energy stored since this one is related to the electron temperature through


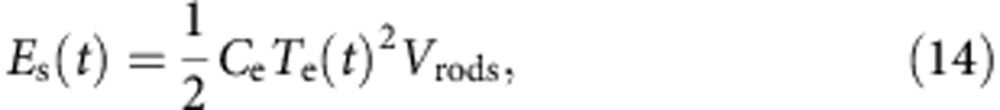


where *C*_e_ is the heat capacity of the electron gas and *V*_rods_ is the total volume of the rods under excitation and depends on the beam diameter. Equation (14) assumes a homogeneous electron temperature in the nanorod and a short exciting pulse such that the electron temperature is not reduced via electron–phonon scattering processes while the pulse propagates through the nanorods. Since these processes are significant at a timescale of few picoseconds, under femtosecond pulse excitation this assumption holds^8^. Equation (13) is solved numerically in the time domain calculating the term *A*(*E*_s_(*t*)) with the transfer matrix method using equations (8, 9, 10, 11) to calculate the intensity-dependent effective refractive index of the nanorod array. From these results, both the nonlinear refraction and absorption can be obtained (Figs 3e,f and 4e,f).

### Numerical modelling of the nonlinear optical response of metamaterial

The optical properties of the plasmonic metamaterial were simulated using the finite element method (FEM, Comsol Multiphysics 4.3a), which accounts for the composite structure of the metamaterial. Furthermore, to also account for the non-homogeneous distribution of the electron temperature in the rods under optical excitation, the two-temperature model^8^ is also solved through FEM together with Maxwell's equations. This model relates the phonon temperature (*T*_L_) and the electron temperature (*T*_e_) through:


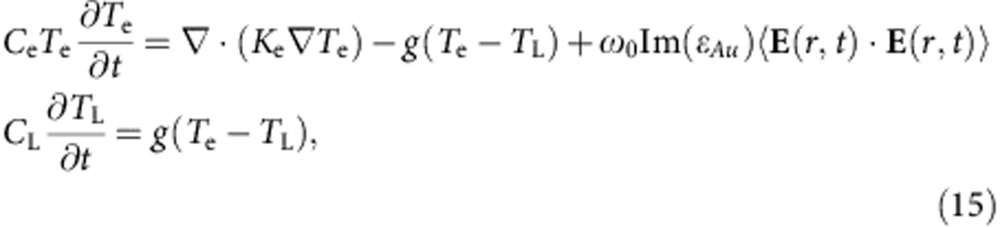


where *C*_e_=67.96 Jm^−3^ K^−2^ is the heat capacity of electrons, *g*=2 × 10^16^ Wm^−3^ K^−1^ is a constant related to the coupling between electrons and phonons, 〈**E**(*r*,*t*)·**E**(*r*,*t*)〉 is the average over time of electric field, the *K*_e_=*K*_e0_*T*_e_*T*_e_/*T*_L_ is the electron heat diffusion with *K*_e0_=318 Wm^−1^ K^−1^ and *C*_L_ is the heat capacity for a lattice^41^. The results obtained using FEM for the nonlinear absorption and refraction are shown in (Figs 3c,d and 4c,d).

### Analysis of the effective *χ* ^(3)^ susceptibility tensor of the metamaterial

Due to the anisotropic nature of the metamaterial, which acts as an uniaxial crystal with the extraordinary axis along the nanorods, the effective permittivity and thus nonlinear susceptibility are tensors. Therefore, the measured effective angular-dependent nonlinear refractive index and absorption can be expressed via the angular-independent components of the nonlinear susceptibility tensors in the similar manner as the angular-dependent permittivity is presented by equation (8). The intensity-dependent complex refractive index of the nanorod metamaterial can be analysed by the effective *χ*^(3)^ susceptibility. The displacement field is then^1^


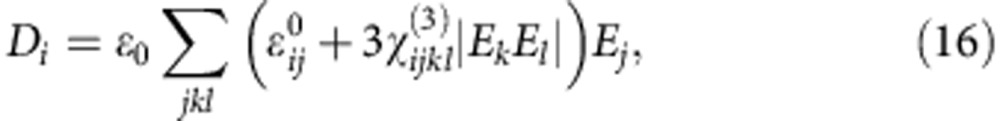


where *ɛ*_0_ is the vacuum permittivity, *E*_*j,k,l*_ is the component of the electric field amplitude in *j*, *k* or *l* directions and is the complex linear permittivity. Equation (16) can be rewritten as 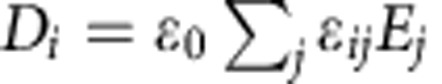, where


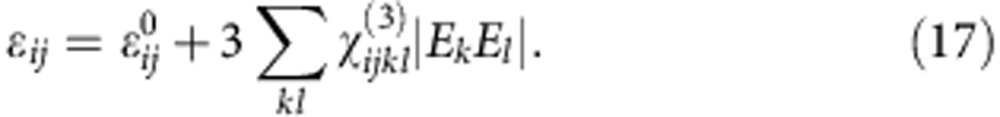


In our case, the linear anisotropic components are *ɛ*_*xx*_=*ɛ*_*yy*_=*ɛ*_*x*_≠*ɛ*_*zz*_=*ɛ*_*z*_, and the incident wave is TM polarized. Therefore, equation (17) becomes


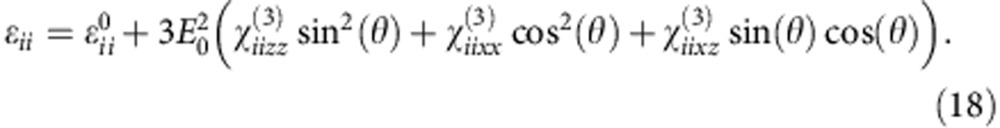


In the case of the elliptic dispersion of the metamaterial for which there is a weak angular dependence for both *γ* and on the angle of incidence observed in the experiment and numerical modelling, we can conclude that both the contributions from both 
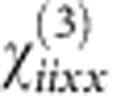
 and 
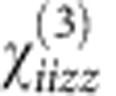
 are present, since the sum of these contributions has a vanishing angular dependence. However, in the ENZ and hyperbolic regimes, the nonlinear coefficients strongly depend on the angle, and the main contribution comes from component 
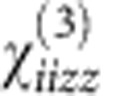
. The contribution of components 
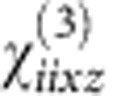
 is negligible, since the angular dependences of *γ* and *β* do not follow sin(2*θ*) dependence. Thus, the dominating effective nonlinear anisotropic tensor components correspond to


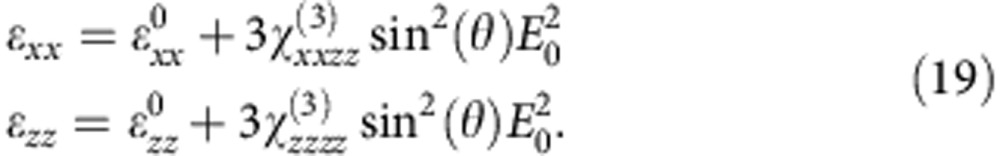


Replacing in equation (8) each nonlinear anisotropic component with equation (19), we obtain the effective nonlinear permittivity


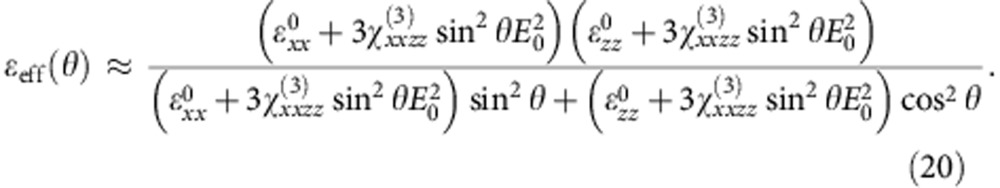


To a first approximation, we can assume that the nonlinear terms in the denominator of equation (20) can be negligible in comparison with the sum of the linear terms and neglect the terms in order of (*E*_0_)^4^:


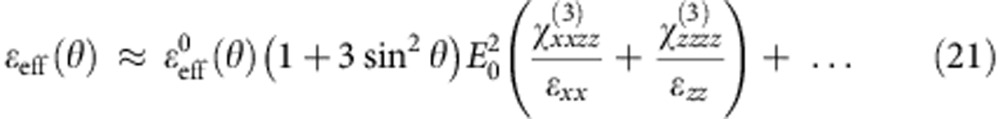


We can make an additional approximation assuming again that the nonlinear terms are small compared with the linear term and, thus, Snell's law can be written linearly as 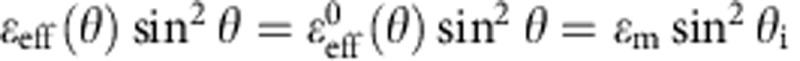, where *ɛ*_m_ is the permittivity of the medium where the incident wave is coming from and *θ*_i_ is the angle of incidence. Therefore, we can rewrite equation (21) as


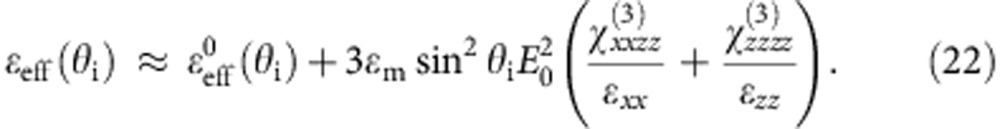


One can observe that the peculiarity of the linear anisotropy can strongly enhance the effective nonlinearity, particularly if a linear permittivity component approaches zero. Equation (22) provides an understanding of the strong enhancement of the metamaterial's optical nonlinearity near the ENZ wavelength (Figs 3 and 4).

## Additional information

**How to cite this article:** Neira, A.D. *et al.* Eliminating material constraints for nonlinearity with plasmonic metamaterials. *Nat. Commun.* 6:7757 doi: 10.1038/ncomms8757 (2015).

## Supplementary Material

Supplementary Information Supplementary Figure 1.
