# Supplementary material for: Eliminating material constraints for nonlinearity with plasmonic metamaterials
Source: Nat Commun. 2015 Jul 21;6:7757. doi: 10.1038/ncomms8757 (PMC4518246; doi:10.1038/ncomms8757)
Supplement: Supplementary Information — Supplementary Figure 1. [file ncomms8757-s1.pdf]

Supplementary Figures

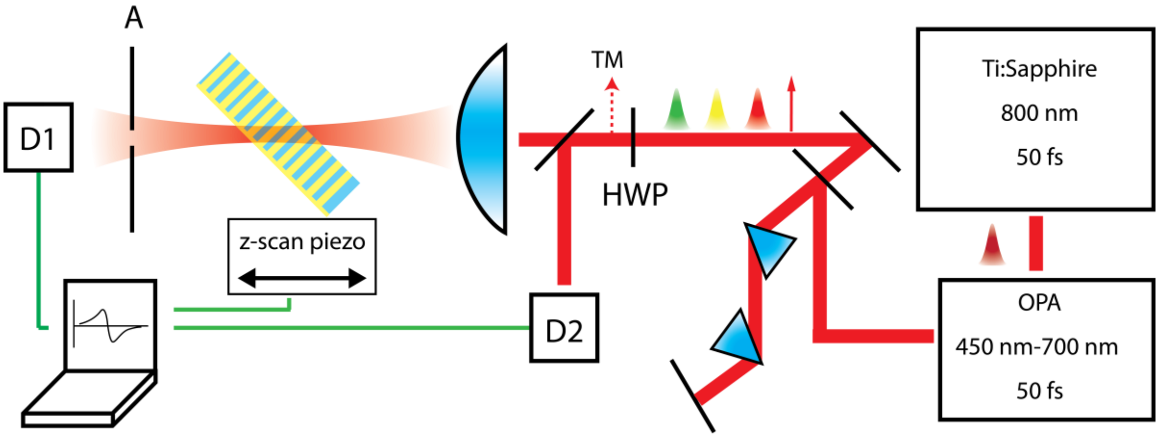

Supplementary figure 1. Z-scan experiment schematic. Light from the OPA pumped with the amplified Ti:sapphire laser illuminates the sample after passing through the prism-based pulse compression system. The half-wave plate HWP controls the light polarization used in the experiment. The aperture A can be open or closed depending on whether an open-aperture or closed-aperture z-scan is performed. D1 and D2 are the InGaAs photodiodes having a bandwidth of 500−1700 nm to measure the transmission through the sample. The sample is placed on a linear piezo-translation stage.
